# Supplementary material for: Profiling of inflammatory cytokines in patients with caustic gastrointestinal tract injury
Source: PLoS One. 2021 Nov 18;16(11):e0260012. doi: 10.1371/journal.pone.0260012 (PMC8601450; doi:10.1371/journal.pone.0260012)
Supplement: Attachment — Submitted filename: Response letter.doc [file pone.0260012.s001.doc]

Journal Requirements

We note that some of the participants in your study had psychiatric comorbidities. To ensure your study complies with our guidelines for human subject research, please include the following information in your ethics statement in the manuscript Methods:

* How were subjects assessed for capacity to consent? Was this done by the researchers or by medical/psychiatric staff? By capacity to consent, we mean the ability to understand and act on a decision, which may not be possible if their mind is impaired or disturbed in some way.
* Were there any inclusion/exclusion criteria based on mental capacity?
* Was participation voluntary, and could they opt out at any time?
* Were subjects undergoing treatment at the time of the study? Did participation in the study affect patient access to treatment?
* Did the ethics committee approve your study protocol and consent procedure knowing that some of the participants that some patients may have psychiatric comorbidities?

Response: Thank you for reminding us. The Ethical statement and Patient recruitment - inclusion and exclusion criteria have been revised.

Ethical statement

This study adhered to the Declaration of Helsinki and had been approved by the Medical Ethics Committee of Chang Gung Memorial Hospital. The Institutional Review Board number allocated to the study was 201602045B0. Informed written consent was obtained from all patients according to the guidelines of our institutional review board. Since some of the patients had psychiatric comorbidities, all patients were routinely assessed for capacity to consent by the principal investigator (H.-T.C.). Patients who refused to sign an informed consent or who had impaired capacity to consent were excluded from analysis. The participation in this study was voluntary and patients can opt out at any time. None of the patients underwent treatment at the time of the study. Furthermore, participation in this study did not affect patient access to treatment. The Medical Ethics Committee of Chang Gung Memorial Hospital had approved the study protocol and consent procedure and knowing that some of the patients may have psychiatric comorbidities.

Patient recruitment - inclusion and exclusion criteria

Between March 2018 and October 2018, we prospectively enrolled 22 patients, including 12 men and 10 women in Chang Gung Memorial Hospital. After discussion with the patient and their family, we performed the procedure. Patients who refused to sign an informed consent or who had impaired capacity to consent were excluded from this study. All patients underwent esophagogastroduodenoscopy with blood serum collection within 24 hours after admission. The mucosal damage was graded using Zagar's modified endoscopic classification scheme. Patients were divided into two subgroups according the mucosal severity of Zagar's classification: mild group (< 2b) and severe group (≥ 2b). Laboratory data, including hematology and biochemistry, were collected upon arrival to the emergency department. The ingested caustic compounds were confirmed by referring to the label on containers. Strong caustics were defined as substances with pH < 2 or > 12. The obtainment of ingested dose and amounts of caustic compounds, intent of ingestion, psychiatric co-morbidities, previous suicide attempt records, treatment courses, intensive care unit admittance, and gastrointestinal/systemic complications were recorded for each case.

Journal Requirements:

1. Point: Please ensure that your manuscript meets PLOS ONE's style requirements, including those for file naming. The PLOS ONE style templates can be found at 
https://journals.plos.org/plosone/s/file?id=wjVg/PLOSOne_formatting_sample_main_body.pdf and 
<https://journals.plos.org/plosone/s/file?id=ba62/PLOSOne_formatting_sample_title_authors_affiliations.pdf>

Response: Thank you for the comment. We have followed the instructions.

2. Point: Please describe in your methods section how capacity to consent was determined for the participants in this study. Please also state whether your ethics committee or IRB approved this consent procedure. If you did not assess capacity please briefly outline why this was not necessary in this case.

Response: Thank you for the comment.

Ethical statement

This study adhered to the Declaration of Helsinki and had been approved by the Medical Ethics Committee of Chang Gung Memorial Hospital. The Institutional Review Board number allocated to the study was 201602045B0. Informed written consent was obtained from all patients according to the guidelines of our institutional review board. Since some of the patients had psychiatric comorbidities, all patients were routinely assessed for capacity to consent by the principal investigator (H.-T.C.). Patients who refused to sign an informed consent or who had impaired capacity to consent were excluded from analysis. The participation in this study was voluntary and patients can opt out at any time. None of the patients underwent treatment at the time of the study. Furthermore, participation in this study did not affect patient access to treatment. The Medical Ethics Committee of Chang Gung Memorial Hospital had approved the study protocol and consent procedure and knowing that some of the patients may have psychiatric comorbidities.

3. Point: We note that you have indicated that data from this study are available upon request. PLOS only allows data to be available upon request if there are legal or ethical restrictions on sharing data publicly. For information on unacceptable data access restrictions, please see http://journals.plos.org/plosone/s/data-availability#loc-unacceptable-data-access-restrictions.

In your revised cover letter, please address the following prompts:

a) If there are ethical or legal restrictions on sharing a de-identified data set, please explain them in detail (e.g., data contain potentially identifying or sensitive patient information) and who has imposed them (e.g., an ethics committee). Please also provide contact information for a data access committee, ethics committee, or other institutional body to which data requests may be sent.

b) If there are no restrictions, please upload the minimal anonymized data set necessary to replicate your study findings as either Supporting Information files or to a stable, public repository and provide us with the relevant URLs, DOIs, or accession numbers. Please see http://www.bmj.com/content/340/bmj.c181.long for guidelines on how to de-identify and prepare clinical data for publication. For a list of acceptable repositories, please see http://journals.plos.org/plosone/s/data-availability#l oc-recommended-repositories.

Response: Thank you for the comment. All relevant data are within the manuscript.

Reviewer #1: Authors have made a good attempt to find the role of interleukins in caustic injury of GI tract. I have following comments to make:

Point 1. Small sample size. How the study subjects were selected since both the groups (mild and severe) had equal number of patients.

Response: Thank you for the comment. All patients with caustic gastrointestinal tract injury seen at our hospital between March 2018 and October 2018 were included in the study. Patients who refused signed informed consent were excluded from the study. The equal number of patients of both groups was by coincidence.

Point 2. Study was done in 2018 why it was not published in last 3 years.

Response: Thank you for the comment. We apologize for the delay in manuscript preparation.

Point 3. Abbreviation of interleukin (IL) can be used instead of repeating it several times.

Response: Thank you for the comment. The term has been abbreviated.

Point 4. What was the aim/objective of the study?

Response: Thank you for the comment. This study aimed to investigate the cytokine profiling of patients with caustic substance ingestion, and analyze the differences between patients with severe and mild caustic gastrointestinal tract injury.

Point 5. Kindly mention the aim in abstract as well.

Response: Thank you for the comment. The aim of this study has been included in the Abstract.

Point 6. Modify table 1. It is confusing as the authors have used "/"and several numbers are written in a single column. This will confuse the readers.

Response: Thank you for the comment. Table 1 has been revised.

Point 7. Authors should mention whether the study is cross-sectional or follow up study. They have vaguely mentioned in discussion and conclusion that it was a short follow up but the details on how the follow up was done and what was the outcome in each patients. They have mentioned that 2 patients died in 3-6 months which is very vague sentence and questions the credibility of the follow up.

Response: Thank you for the comment. We confirmed that this is a cross-sectional study. We apologize for the vague sentence, the inappropriate sentence has ben revised.

Point 8. Mention some more details regarding the type of surgery /indication/ timing etc in these patients.

Response: Thank you for the comment. A new Table 5 has been created to describe the clinical courses of the 5 patients who underwent surgery. The indications for operations were mainly esophageal stricture or gastric outlet obstruction.

Point 9. What is the implication of this study on the treatment of patients as it is mentioned that steroids have no role in the treatment. How will the patients be benefitted by measuring the IL levels?

Response: Thank you for the comment. None of our patients were treated with glucocorticoids. Beneficial effects of steroid on preventing stricture formation in severe caustic ingestion, is controversial according to published literatures. Nevertheless, the findings of this translational study provide clinical significance. Our analysis found that caustic gastrointestinal tract injury is characterized by a systemic inflammatory response that involves elevated levels of circulating inflammatory cytokines and neutrophils. The analysis showed increased circulating levels of inflammatory cytokines (IL-2, IL-5, IL-8, IL-9, IL-12, IL-13, interferon-gamma inducible protein-10, macrophage inflammatory protein-1 beta, regulated upon activation, normal T cell expressed and presumably secreted and tumor necrosis factor-alpha) in patients with caustic gastrointestinal injury than healthy controls. Furthermore, the neutrophil counts and circulating levels of certain cytokines (IL-2 and tumor necrosis factor-alpha) were higher in patients with severe than mild caustic gastrointestinal injury. Additionally, since circulating levels of IL-2 and tumor necrosis factor-alpha were higher in nonsurvivors than in survivors, these two cytokines might have clinical potential as promising prognostic markers for caustic gastrointestinal injury. In this context, clinical determination of the circulating inflammatory response, particularly IL-2 and tumor necrosis factor-alpha levels, could serve as a valuable adjunct to physiological predictors for the prediction of poor outcome.

Point 10. Mention the novelty of study and its role in future research.

Response: Thank you for the comment. This is supposed to be the first study to profile inflammatory cytokines in patients with caustic gastrointestinal tract injury. Caustic injury of the upper gastrointestinal tract causes severe mucosal damages and leads to excessive levels of inflammatory cytokines and neutrophils in the peripheral blood. The findings of this translational study provide clinical significance. Our analysis found that caustic gastrointestinal tract injury is characterized by a systemic inflammatory response that involves elevated levels of circulating inflammatory cytokines and neutrophils. The analysis showed increased circulating levels of inflammatory cytokines (IL-2, IL-5, IL-8, IL-9, IL-12, IL-13, interferon-gamma inducible protein-10, macrophage inflammatory protein-1 beta, regulated upon activation, normal T cell expressed and presumably secreted and tumor necrosis factor-alpha) in patients with caustic gastrointestinal injury than healthy controls. Furthermore, the neutrophil counts and circulating levels of certain cytokines (IL-2 and tumor necrosis factor-alpha) were higher in patients with severe than mild caustic gastrointestinal injury. Additionally, since neutrophil counts and circulating levels of IL-2 and tumor necrosis factor-alpha were higher in nonsurvivors than in survivors, these two cytokines might have clinical potential as promising prognostic markers for caustic gastrointestinal injury. In this context, clinical determination of the circulating inflammatory response, particularly IL-2 and tumor necrosis factor-alpha levels, could serve as a valuable adjunct to physiological predictors for the prediction of poor outcome.

Reviewer #2: This study analysis cytokine response after caustic gastrointestinal injury, and though the change in cytokine level might be the biomarker for outcome prediction. It showed elevation of such as interleukin (IL) 2,5, 8, 9, 12 interferon gamma, and tumor necrosis factor (TNF) alpha after caustic injury compaired with healthy controls. Especially IL-2 and TNF-alpha were higher in severe caustic injury group. But there is no further analysis for cytokine change in complication such as systemic complication, or GI complication such as stricture. It is hard to conclude that there is no benefit of steroid for prevent esophageal stricture owing to no significance difference in IL-10 in caustic injruy group. One study had analysis Infliximab, TNF alpha antibody, in experimental alkali burns of the oesophagus in the rat. Knowing the cytokine response related complication after caustic injury may evoke more study for treatment.

Response: Thank you for the comment. We are agreed that this study is limited by small sample size and lacking serial cytokine measurement. This important limitation has been included to the Discussion section.

The most obvious limitation in this study was small sample size. Moreover, there was no further analysis for cytokine change in complication such as systemic complication, or gastrointestinal complication such as stricture. Therefore, it was difficult to conclude that there is no benefit of steroid for prevent esophageal stricture owing to no significance difference in IL-10 between two groups of caustic patients. Notably, anti-tumor necrosis factor alpha treatment with infliximab did not ameliorate the degree of fibrosis in alkali burns of the esophagus in the rat. Knowing the cytokine response related complication after caustic injury may evoke more study for treatment. Further studies are necessary to elucidate the influences of immune responses on the clinical outcomes of corrosive patients.
